# Adipose-Derived Mesenchymal Stromal/Stem Cells: Tissue Localization, Characterization, and Heterogeneity

**DOI:** 10.1155/2012/812693

**Published:** 2012-04-12

**Authors:** Patrick C. Baer, Helmut Geiger

**Affiliations:** Division of Nephrology, Department of Internal Medicine III, Johann Wolfgang Goethe University, 60590 Frankfurt, Germany

## Abstract

Adipose tissue as a stem cell source is ubiquitously available and has several advantages compared to other sources. It is easily accessible in large quantities with minimal invasive harvesting procedure, and isolation of adipose-derived mesenchymal stromal/stem cells (ASCs) yields a high amount of stem cells, which is essential for stem-cell-based therapies and tissue engineering. Several studies have provided evidence that ASCs in situ reside in a perivascular niche, whereas the exact localization of ASCs in native adipose tissue is still under debate. ASCs are isolated by their capacity to adhere to plastic. Nevertheless, recent isolation and culture techniques lack standardization. Cultured cells are characterized by their expression of characteristic markers and their capacity to differentiate into cells from meso-, ecto-, and entodermal lineages. ASCs possess a high plasticity and differentiate into various cell types, including adipocytes, osteoblasts, chondrocytes, myocytes, hepatocytes, neural cells, and endothelial and epithelial cells. Nevertheless, recent studies suggest that ASCs are a heterogeneous mixture of cells containing subpopulations of stem and more committed progenitor cells. This paper summarizes and discusses the current knowledge of the tissue localization of ASCs in situ, their characterization and heterogeneity *in vitro*, and the lack of standardization in isolation and culture methods.

## 1. Introduction: Mesenchymal Stromal/Stem Cells

The pathologist Cohnheim first observed the presence of nonhematopoietic stem cells in the bone marrow in 1867 [1]. He hypothesized that cells with a fibroblast-like morphology migrate to the sites of injury and help to regenerate damaged tissue. The pioneering work of Friedenstein and coworkers in the 1960s [2, 3] on the isolation, culture, and osteogenic differentiation of bone-marrow-derived cells opened a new field of stem cell research. Nearly 20 years later, Owen [4] and Caplan [5] introduced the terms stromal stem cells and mesenchymal stem cells (MSCs) to the scientific community. Whereas, in these initial works, MSCs were isolated from adult bone marrow, in the next decades, MSCs were also found in nearly all adult tissues (e.g., adipose tissue, synovium, dermis, periosteum, deciduous teeth), in peripheral blood, menstrual blood, and in solid organs (e.g., liver, spleen, lung) [6–8]. MSCs are a rare and quiescent population in their niche within fully specialized tissues. At present, there is a strong amount of data indicating that MSCs represent independent population(s) of stem cells with self-renewal properties and established multipotent differentiation profile *in vitro* [9]. Furthermore, MSCs are attractive candidates for clinical applications to repair or regenerate damaged tissues, especially because these cells hold no ethical concerns and can be isolated in appropriate amounts from several sources and proliferated in culture. In addition, MSCs from autologous origin seem to be a safe source for cell-based regenerative approaches.

There is also evidence that MSC preparations are heterogeneous cell cultures comprising a subset of stem cells (or different subsets of stem cells) and more differentiated (progenitor) cells. To address the inconsistency between the nomenclature and biologic properties of this heterogeneous population, the International Society for Cellular Therapy has suggested that these plastic-adherent cells, regardless of the tissue from which they are isolated, be termed multipotent mesenchymal stromal cells, while the term mesenchymal stem cells should be used only for the subset (or subsets) that meets specified stem cell criteria [10]. In general, MSCs are isolated by their capacity to adhere to culture-dish plastic. The cells can be expanded in culture while maintaining their multipotency during standard cell culture and are immunologically characterized by a specific panel of markers. However, the characterization of MSCs remains difficult due to the lack of a definitive and unique cellular marker. Therefore, the International Society for Cellular Therapy proposed three minimal criteria for the definition of cultured MSCs: (a) plastic adherence, (b) expression of CD73, CD90, and CD105, and lack of CD11b or CD14, CD 19 or CD79*α*, CD45, and HLA-DR expression, and (c) their trilineage differentiation potential into adipocytes, chondrocytes, and osteoblasts [11]. Furthermore, MSCs have reduced immunogenic properties and an immunosuppressive potential, which make them also attractive for allogenic stem cell therapy [12–15]. In addition, ideal MSCs for use in therapeutical approaches need to be isolated with minimal harm for the patient, must be available in high cell numbers, proliferate in culture, and differentiate into a broad spectrum of lineages.

## 2. Adipose-Derived Mesenchymal Stromal/Stem Cells

Over the past ten years, it has been recognized that fat is not only an energy reservoir, but also a rich source of multipotent stem cells. Subcutaneous adipose depots are ubiquitous and easily accessible in large quantities with a minimal invasive procedure (by liposuction aspiration). Liposuction surgery is a well-tolerated and safe procedure yielding large quantities of aspirate. The method is cheaper and less invasive than bone marrow aspiration for stem cell isolation. Furthermore, the lipoaspirate is finally discarded as medical waste, qualifying this starting material as a good source of (autologous) adipose-derived mesenchymal stromal/stem cells (ASCs) for further cell isolation. Nevertheless, it is also possible to isolate ASCs from needle biopsies of human adipose tissue or from inguinal fat pads in mice, as well as from other mammals [16–20].

Adipose tissue contains a large number of multipotent cells, which is an essential prerequisite for stem-cell-based therapies. It has been described that stem and progenitor cells in the uncultured stroma-vascular fraction (SVF) from adipose tissue usually amount to up to 3% of the whole cells, and this is 2,500-fold more than the frequency of stem cells in bone marrow [21]. Others have also described that adipose tissue provides large numbers of stem cells compared to bone marrow. A bone marrow transplant contains approximately 6 × 10^6^ nucleated cells per mL [22], of which only 0.001–0.01% are stem cells [23]. In comparison, the number of SVF cells that can be isolated from subcutaneous liposuction aspirates is approximately 0.5–2.0 × 10^6^ cells per gram of adipose tissue [22, 24–27], whereby the percentages of stem cells range from 1 to 10% [26, 28, 29], most likely depending on the donor and tissue harvesting site. Therefore, approximately 0.5 × 10^4^ to 2 × 10^5^ stem cells can be isolated per gram of adipose tissue, varying among patients.

The isolation method and the cytological characterization of stromal precursor cells from adipose tissue were shown at the beginning of the 1970s [30, 31]. Later, the adipogenic and osteogenic potential of this cell population was analyzed [32–35]. The work of Zuk and coworkers in 2001 and 2002 first characterized the multipotent character of ASCs [24, 36]. It should be mentioned that a different nomenclature for the isolated cell population was used in the literature, thus generating a confusing discrepancy. The terms “adipose-derived adult stem (ADAS) cells,” “adipose-derived adult stromal cells,” “adipose-derived stromal cells (ADSC),” “adipose stromal cells (ASC),” “adipose mesenchymal stem cells (AdMSC),” “preadipocytes,” “processed lipoaspirate (PLA) cells,” and “adipose-derived stromal/stem cells (ASCs)” for cells isolated almost by a similar isolation procedure (plastic adherence) and, therefore, probably the same cell population(s) were used in the literature. To eliminate this discrepancy, the International Fat Applied Technology Society (IFATS) reached a consensus to adopt the term “adipose-derived stromal/stem cells” to identify the plastic-adherent, cultured and serially passaged, and multipotent cell population from adipose tissue [28, 37].

## 3. Origin of ASCs In Situ

Several studies have tried to identify the location of the stem cell population within intact adipose tissue. This is a complicated endeavour because no single marker specifically and unequivocally identifies undifferentiated ASCs (as well as MSCs in general). Results from these histological studies using immunohistochemical and immunofluorescence techniques suggest that a stem cell population (or populations) resides in a perivascular location, where ASCs coexist with pericytes and endothelial cells. It has also been suggested that ASCs (and MSCs in general) are a subset of pericytes or vascular stem/precursor cells at various stages of differentiation located in the wall surrounding the vasculature [38]. It has also been hypothesized that blood vessels in virtually all organs and tissues harbour ubiquitous (mesenchymal) stem cells in their perivascular niche [39].

Several investigations encouraged the hypothesis of a perivascular localization of ASCs. It has been speculated that ASCs exist as CD34^+^/CD31^−^/CD140*β* ^−^/*α*-smooth muscle actin^−^ (smA) cells in capillaries and in the adventitia of larger vessels [40]. Zimmerlin and coworkers investigated the localization of known endothelial and perivascular markers in sections of intact adipose tissue and detected a CD90^+^/CD34^+^/CD31^−^/CD146^−^/smA^−^ population in the outer adventitial ring of the vasculature [41]. They identified these cells as supra adventitial ASCs. Traktuev and coworkers described that ASCs are primarily located in the walls of adipose microvasculature in a CD34^+^/CD31^−^ phenotype [42]. Another study of this group described that these cells are capable of stabilizing endothelial networks *in vitro*, as well as robustly synergizing with endothelial cells to participate in the *in vivo* formation of new vessels which connect with host vasculature, conduct blood flow, and exhibit network stability for several weeks [43]. Others described a perivascular cell subset in the smallest blood vessels and adventitial cells around larger ones, which natively expresses mesenchymal stem cell markers and displays multilineage differentiation in culture [39, 44, 45]. The authors identified these perivascular cells by their expression of CD146, neuroglial proteoglycan 2 (NG2), and CD140*β*, in addition to standard MSC markers (CD44, CD73, CD90, CD105). Nevertheless, the cell subset described did not express CD34. In addition, others described CD146^+^ cells in the perivascular region that exhibit the biological properties of MSCs if isolated and cultured [46]. Therefore, due to the expression of CD146, these cells are clearly distinct from the cells described by Lin, Zimmerlin, and Traktuev. There is also another recent work, in contrast to these studies, which described that only the smA^+^ cells from murine adipose tissue display a multilineage differentiation potential, while smA^−^ cells only differentiate into adipocytes *in vitro* [47].

Whereas all these studies provided much evidence and it seems likely that ASCs in situ reside in a perivascular niche in a CD34^+^/CD90^+^/CD31^−^/CD45^−^/CD146^−^ phenotype, the definite identification of the ASC population(s) in situ has currently not been achieved. The niche (local microenvironment) is a crucial determinant not only of stem cell fate, function, and maintenance, but maybe also of the ASCs' phenotype.

## 4. Characterization of Uncultured Primary Isolates

ASCs can easily be isolated by tissue digestion and centrifugation steps, followed by the outgrowth of the plastic adherent fraction from the primary isolated cell mixture (the so-called SVF) [24]. SVF is a highly heterogeneous cell population, because it also comprises the nonadherent cell population. The composition of the SVF has been reported with great variability among authors. Cell populations within the SVF could be roughly distinguished by cell size and granularity in flow cytometry by forward and sideward scatter diagrams and by their characteristic expression pattern. Miranville and coworkers described some stem cell markers (CD34, CD133, ABCG2) in the SVF from different anatomic sources. They first described that freshly harvested SVF contains large numbers of CD34^+^ cells and showed two subpopulations of CD34^+^ cells [48]. A more comprehensive characterization was done by Yoshimura and coworkers. They identified cell populations in the SVF including the following potential ASCs (CD31^−^/CD34^+^/CD45^−^/CD90^+^/CD105^−^/CD146^−^), endothelial (progenitor) cells (CD31^+^/CD34^+^/CD45^−^/CD90^+^/CD105^low^/CD146^+^), pericytes (CD31^−^/CD34^−^/CD45^−^/CD90^+^/CD105^−^/CD146^+^), and blood-derived cells (CD45^+^) by multicolour flow cytometric analysis [49], whereas it is most likely that also fibroblasts, vascular smooth muscle cells, and preadipocytes are present in the SVF. It has also been described that the SVF is composed of 11% CD2^+^ cells, 18% CD11a^+^ cells, 29% CD14^+^ cells, 49% CD31^+^ cells, 57% CD45^+^ cells, and 60% CD90^+^ cells (referring to ASCs and endothelial cells) [50]. Others detected a different composition of the SVF (nearly 11% CD14^+^ cells, ~2% CD31^+^ cells, ~7% CD34^+^, ~9% CD45^+^ cells, ~29% CD90^+^, and ~47% 146^+^ cells) [51].

It has been demonstrated that more than 85% of the SVF cells that initially adhered to the culture wells had a CD31^−^/CD34^+^/CD45^−^/CD146^−^ phenotype [52]. Within the CD34^+^ cells, two subpopulations with different phenotypes have been identified (a CD34^dim^ and CD34^bright^ subpopulation) [51]. In addition, it has been described that the CD31^−^/CD34^+^/CD45^−^/CD105^+^ cells from purified uncultured adipose tissue display stem cell properties [53]. The authors also compared CD31^−^ and CD31^+^ cells from the SVF and showed that only the CD31^−^ subpopulation displayed multilineage differentiation *in vitro*. Nevertheless, the data currently available are inconsistent and not adequate for the clear definition of an exclusive ASCs population in the SVF. Sharing membrane antigens with other cells found in the SVF, ASCs could not be definitely distinguished in the whole heterogeneous SVF cell mixture. This is indeed also based on two facts: (1) that there are several stem cell subpopulations within the SVF and (2) that the cells are related to the plastic adherent and cultured population which dramatically changes the phenotype very early during cell culture.

## 5. Characterization and Heterogeneity of Cultured ASCs

The fraction of adherent cells cultured in standard cell culture medium is considered as multipotent ASCs. The cells of this fraction are characterized early during primary culture by a slightly heterogeneous morphology indicating different stem and precursor cell subpopulations and (maybe) more differentiated cells (dedifferentiated endothelial cells, smooth muscle cells, and pericytes). Heterogeneity of MSC isolations in general has been discussed in many publications [54–57]. Nevertheless, when analyzing the adherent population by flow cytometry, no macrophages, endothelial cells, lymphocytes, or granulocytes seem to remain [50]: for example, the presence of endothelial cells is not detectable [50]. However, endothelial cells in culture are extremely susceptible to culture conditions, such as supplements and particularly shear stress, and, therefore, may dedifferentiate or trigger apoptosis under static culture conditions [57].

The heterogeneity of cultured ASCs can be reduced by a washing procedure early in the beginning of the cell culture [58], indicating that several subsets require different time points to adhere to the cell culture plastic. Other efforts to reduce the heterogeneity or to isolate specific subsets of ASCs were carried out by using flow cytometric sorting or immunomagnetic separation, either by positive or by negative selection [59–62]. The usage of such techniques for the reduction of heterogeneity is more or less beneficial but leads to a very small cell yield. By using immunomagnetic beads, Rada and coworkers demonstrated that the SVF is composed of several subpopulations, which express different levels of ASC markers and exhibit varying osteogenic and chondrogenic differentiation potentials [60].

Cultured ASCs show an extensive proliferative ability in an uncommitted state while retaining their multilineage differentiation potential. In later passages, ASC cultures are homogeneous and exhibit a fibroblastoid morphology. The composition of subpopulations, therefore, may change during expansion [63]. Cell culture selects for this homogeneous morphology, enriching for cells expressing a stromal immunophenotype [28]. Different studies have characterized and compared the immunophenotype of cultured ASCs in early and later passages over the past few years and found that the expression profile of ASCs changes during culture time. It has been repeatedly shown that freshly isolated ASCs express different surface markers than ASCs in higher passages [28, 52]. At the beginning of the culture, ASCs do not uniformly express all surface proteins, which are supposed to be characteristic. Subsets with distinct phenotypic properties can be discerned in freshly isolated cells by specific surface markers [63]. On the other hand, ASCs in passage 2 or 3 uniformly express their characteristic markers (positive for CD10, CD13, CD29, CD 44, CD49e, CD73, CD90, CD105, and CD166, and negative for CD11b, CD14, CD31, and HLA-DR) [35]. The expression of the markers seems to be dependent on culture conditions or time in culture. The specific surface markers CD29, CD90, and CD166 increase during culture [28], while the expression of other markers decreases [28, 52]. Similar to SVF and contrary to long-term cultured ASCs, freshly isolated ASCs are described as expressing CD34, CD117, and HLA-DR [52]. In the case of CD34, it has been demonstrated that more than 95% of the cells are still CD34 positive after one week of culture, whereas, subsequently, the expression level of CD34 decreases dramatically during culture. It has also been described that only some ASCs lose their CD34 expression with increasing culture time and that cell culture in medium 199 supplemented with acidic FGF maintained CD34 expression for at least 10–20 weeks [49]. On the other hand, expression of CD105 and especially CD166 is relatively low on the freshly isolated ASCs but rises to a high extent during cell culture [52]. Another study also described that stromal cell-associated markers (CD29, CD73, CD166) are initially expressed lower but rise during successive passages, whereas the expression of CD34 dramatically decreases [28].

Whereas expression of some characteristic markers is consistently found to be expressed by cultured ASCs and others are consistently not found to be expressed (summarized in [64]), many studies differ in some of the markers. The expression of some antigens is described in a very contrary way. Some reports described CD34, CD54, CD107, or CD146 to be expressed on cultured ASCs, and others did not find the expression of these antigens. These differing results are due to differences in the isolation or culture method or caused by the investigation of different passages of cultured ASCs: for example, although CD34 is reckoned as a hematopoietic stem-cell-associated marker, it is expressed by early passages of ASCs' subsets and subsequently lost in later passages [28, 49]. Another study compared the CD34^+^ and CD34^−^ subsets of ASCs and found that CD34^+^ cells are more proliferative and have a higher ability to form colonies, while CD34^−^ cells have a greater ability to differentiate into adipogenic and osteogenic lineages [65]. CD34^+^ cells also expressed other endothelial markers, whereas CD34^−^ cells expressed markers such as CD146. CD146 is described as lowly expressed by the whole ASCs population, and this expression decreases with culture time [28]. Since CD146 is also a marker for endothelial cells and pericytes, it could belong to a subset of ASCs [26, 49]. Taken together, comprehensive studies are needed to further characterize the whole expression profile of ASCs in different passages in detail.

## 6. What about Standardization of the Isolation and Culture Procedure?

Discrepancies in the results of studies from different laboratories may result from many different origins. First of all, ASCs are isolated from different donors. These donors differ in age, body mass index, gender, ethnicity, and their medical history (e.g., preexisting diseases, nicotine, or alcohol abuse in humans). It has been shown, for example, that the body mass index correlates negatively to the number of stromal cells per gram and their differentiation capacity [66]. The liposuction procedure may differ between different clinics, the liposuction (or biopsy) side is different, and the time lapse until isolation procedure starts differs between the laboratories. It has been reported for ASCs that liposuction side, liposuction procedure, age, or body mass index play an important role in the cell yield, growth, and frequency of stem cells [26, 66–69], but it is not clear whether this favours different subsets in cultured ASCs. All these variables may affect the composition of the isolated initial cell culture, but it is extremely difficult, if not impossible, to standardize these variables.

On the other hand, the methods and quality of isolations of ASCs from different laboratories per se vary tremendously, resulting in a different composition of the initial cell culture. Finally, the culture procedure of isolated ASCs differs between the laboratories; at the present time, there is no unique and standardized culture protocol for the culture of ASCs. There are many variables that impair the cultured cells (or the composition of subpopulations) in their undifferentiated state: initial plating density and confluency, coating of culture dishes and stiffness of the substrate, composition of cell culture basal media, cell culture supplements (bovine serum, human serum, platelet lysate, or growth factors), addition of antibiotics, oxygen supply (hypoxia), and method of subculturing and cryopreservation (Table 1).

*In vivo*, many cell types are attached to soft materials, either other cells or extracellular matrices, but most of what is known about cell structure and function *in vitro* derives from studies of cells plated onto rigid substrates, such as plastic [70]. As a result, some aspects found *in vitro* are rarely if ever seen *in vivo* [70]. Differentiation of MSCs, for example, has been shown to be dependent on the substrate on which the cells are cultured. Whereas MSCs on stiff substrates expressed markers of osteogenesis, MSCs on softer substrates expressed myogenic markers, and cells on the softest gels expressed neuronal markers [71]. Nevertheless, stiffness alone is not sufficient to fully differentiate cells. Furthermore, the influence of plastic coating with collagen or fibronectin has also been shown to influence the differentiation state of MSCs [72]. Therefore, more work about the optimal substrate and substrate stiffness to culture ASCs is highly desirable.

Only limited information is available about which medium optimally expands ASCs by maintaining the undifferentiated stem cell character *in vitro* [73–75]. It has been shown in cultures of MSCs that basal medium, glucose concentration, quality of FCS, cell plating, and cell density highly affect the final outcome [76], resulting in the expansion of populations with totally different potential. The media composition, for example, highly effects the expression of the stem-cell-related transcription factors NANOG, Oct-4, Sox-2, and Rex-1 in ASCs [75]. These factors have also been shown to be expressed by ASCs in earlier studies [27, 77, 78] and are related to the undifferentiated state of ASCs (and also to pluripotency of stem cells in general). Many laboratories use Dulbecco's modified Eagle medium (DMEM) as a basal medium to culture ASCs, but there are different DMEMs commercially available, and a further description of the exact medium used in these studies is often not indicated. We use DMEM with an approximately physiological glucose content (100 mg/dL). Others use a standard DMEM with a higher glucose content, because, in this medium, ASCs show a much better proliferation rate. Nevertheless, a physiological glucose content is one variable which should be considered to be near to the *in vivo* situation. Furthermore, a low calcium concentration and supplementation with antioxidants have been shown to accelerate the proliferation of ASCs, but it was not clearly shown that this culture medium did not alter the whole differentiation capacity of ASCs [79].

Most of the investigators use DMEM with 10% foetal calf or bovine serum as a standard proliferation medium, whereas others use low-serum expansion media supplemented with one or more growth factors, for example, epidermal growth factor (EGF), platelet-derived growth factor, and/or basic fibroblast growth factor [80–83]. There are many concerns about the practicability of foetal calf or bovine serum (infectious complications, host immune reactions) related to a possible use of ASCs in human therapeutical approaches [84]. Additionally, human serum may be a source of pathogen contamination or immunoreactivity and shows batch-to-batch variability. Using defined cell culture medium is an urgent need in order to produce ASCs for clinical applications. The gold standard for culturing ASCs would be a medium absolutely free of animal serum or factors, with well-known ingredients. Parker and coworkers tested eight commercially available serum-free media developed for use with other cell lines for their ability to support growth of human ASCs [74]. None of the available media was sufficient for supporting cell growth as purchased, and none performed better as a base medium than their standard medium containing serum. Others described serial testing of new medium formulations containing human serum or platelet lysate or tested the use of animal serum- or xeno-free media for the culture of ASCs in regard to cell morphology, cell proliferation, phenotype, and differentiation potential [85–88]. As a result of these studies, there is obviously no favourable serum- and xeno-free medium for the expansion of ASCs retaining their undifferentiated state. Rajala and coworkers, for example, described a xeno-free medium that induced significantly higher proliferation rates than medium containing allogeneic human serum [88]. This medium maintained the differentiation potential of ASCs. Nevertheless, the authors detected significant differences in the surface marker expression of ASCs cultured in xeno-free medium compared with human serum.

In summary, modifications in the isolation and/or culture conditions might select for the expansion of subpopulations and have a huge impact on the differentiation potential of the cells cultured, albeit the primary cells could be phenotypically identical [57]. Therefore, standardization of the isolation and culture procedure is highly needed for a good reproducibility of results from different laboratories and studies.

## 7. The Differentiation Potential of ASCs

The *in vitro* differentiation of ASCs into multiple cell types of mesodermal origin has been shown in a variety of studies. ASCs can be cultured by serial passaging without losing their multipotent properties [27] and have the capacity to maintain chromosome stability in long-term cultures [89]. Different studies described ASCs' plasticity towards chondrocytes, osteoblasts, adipocytes, and myocytes (cardiomyocytes, smooth muscle, and skeletal muscle cells) [24, 36, 90–97]. In general, the induction of ASCs' differentiation *in vitro* is mainly achieved by culture in selective media with lineage-specific induction factors. The transcriptional and molecular events triggering the mesodermal lineage-specific differentiation of stem cells are well known [98–103]. ASCs have also been shown to be angiogenic and hematopoietic supporting cells [104–106]. These supporting characteristics of ASCs are mainly due to the secretion of antiapoptotic angiogenic and hematopoietic factors (cytokines and growth factors), such as macrophage colony-stimulating factor, granulocyte macrophage colony-stimulating factor, insulin-like growth factor, hepatocyte growth factor, vascular endothelial growth factor, hepatocyte growth factor, and transforming growth factor-*β* [107–109].

The potential of ASCs to differentiate into lineages with nonmesodermal origin, although ASCs originate from the mesoderm, is even more exciting. The differentiation potential of ASCs into cells of ecto- and endodermal origin has also been shown. Therefore, the term pluripotent stem cells would be more correct for ASCs (rather than multipotent), as a differentiation into cells from all three germ layers has been shown. Nevertheless, the morphology of ASCs is different to other pluripotent stem cells, and their ability to form teratoma has not been shown. Therefore, ASCs' pluripotency is not accepted overall in the scientific community.

A variety of studies documented the induced *in vitro* differentiation into hepatocytes, pancreatic islet cells, neural cells, endothelial cells, and epithelial cells [16, 50, 75, 110–120]. Our studies have clearly verified that ASCs can enter the epithelial lineage when treated with retinoids [50], conditioned medium (CM) from renal tubular epithelial cells, or a mixture of growth factors [116–119]. *In vivo* differentiation of ASCs toward renal epithelial cells has also been shown in a renal ischaemia-reperfusion model [120]. The multiorgan engraftment of transplanted ASCs has been shown, in combination with epithelial lineage differentiation [121]. Fang and coworkers examined the *in vivo* characteristics and behaviour of human ASCs transplanted in sublethally irradiated nonobese mice with diabetes or severe combined immunodeficiency. They demonstrated that ASCs differentiate into epithelial cells of the gastrointestinal tract, liver, and bronchi, and endothelial cells by using immunofluorescence staining and in situ hybridization.

The multilineage potential of ASCs has also been shown at the single cell level [29, 95, 122]. Clonal analysis of single-cell-derived colonies of MSCs demonstrated that not every cell possesses a trilineage differentiation potential [63, 123]. This is also the case in single cell clones from human ASCs, which were induced for adipogenesis, osteogenesis, chondrogenesis, and neurogenesis using lineage-specific differentiation media [29]. Eighty-one percent of the clones differentiated into at least one of the lineages, and 52% of the clones differentiated into two or more of the lineages. The authors, therefore, reasoned that ASCs are a type of multipotent adult stem cell and not solely a mixed population of unipotent progenitor cells [29]. It has been demonstrated that single clones of ASCs isolated from mouse inguinal fat pads are capable of clonogenic, myogenic, adipogenic, and neurogenic differentiation [122].

Several tissue engineering and cell therapeutical approaches using ASCs with or without scaffolds have been carried out in animal experiments to verify ASCs' *in vitro* differentiation potential. Besides the obvious applications of ASCs to repair or regenerate cartilage, bone, muscle, or adipose tissue, the possibility of peripheral nerve regeneration, hepatic regeneration, insulin-producing islet cell regeneration, functional repair of myocardial infarction, and recovery of renal function has recently been shown in *in vivo* models [124–130]. In most of these *in vivo* studies, undifferentiated ASCs proliferated under conditions preserving the undifferentiated state were transplanted or used to assemble tissue engineered constructs. Nevertheless, some researchers used ASCs predifferentiated in culture [131] or genetically modified ASCs in their *in vivo* models [129, 132, 133].

## 8. Final Remarks

Apart from all the proven basic scientific evidence in *in vitro* and *in vivo* studies which have been accumulated during the last few years, we are at the beginning of a new era of stem cell therapy. ASCs are probably one of the most powerful adult stem cells. Both preclinical studies and clinical trials using ASCs have been initiated for autologous or allogenic therapeutical trials (recently reviewed in [134]). Nevertheless, the lack of standardization in the isolation methods and culture protocols needs to be overcome in order to eliminate the significant variability in cell quality. Research progress has also been hampered by the limited knowledge of ASCs' subsets, due to the lack of unique markers for their isolation. In addition, good manufacturing practices (GMP), qualified isolation, and culture protocols are needed for the use of ASCs in clinical trials [135, 136]. A variety of other questions need to be answered before ASCs can be used in standard clinical usages: bio-safety (tumour capacity), reproducibility, and efficiency of transplanted ASCs. Moreover, additional studies using *in vivo* models are needed to augment our understanding of how the migration, growth, and differentiation of ASCs are governed by interactions with resident cells, growth factors, and cytokines during regeneration or repair. Nevertheless, the entirety of recent *in vivo* studies and of the few published case reports and clinical trials has shown that ASCs are on the direct path to their clinical usage for the treatment of a multitude of diseases.

## Figures and Tables

**Table 1:** Summary of cell culture parameters which affect the undifferentiated state of ASCs.

(i) Basal medium	→ Medium composition (e.g., DMEM, *α*MEM, M199)
	→ Glucose content
	→ Calcium content

(ii) Supplements	→ Serum (bovine or human)
	→ Platelet lysate
	→ Growth factors (e.g., bFGF, aFGF, EGF, PDGF)
	→ Corticoids
	→ Antioxidants
	→ Antibiotics (?)

(iii) Environment	→ Hypoxia
	→ Perfusion culture (Shear stress)
	→ Stiffness of the substrate (coating)
	→ Mechanical strain
	→ Confluency (cell cell-contacts)
